# Dynamic Changes in the Quantitative Electroencephalographic Spectrum During Attention Tasks in Patients With Prader–Willi Syndrome

**DOI:** 10.3389/fgene.2022.763244

**Published:** 2022-03-16

**Authors:** Li-Ping Tsai, Syu-Siang Wang, Siew-Yin Chee, Shi-Bing Wong

**Affiliations:** ^1^ Department of Pediatrics, Taipei Tzu Chi Hospital, Buddhist Tzu Chi Medical Foundation, New Taipei, Taiwan; ^2^ School of Medicine, Tzu Chi University, Hualien, Taiwan; ^3^ Research Center for Information Technology Innovation, Academia Sinica, Hualien, Taiwan

**Keywords:** Prader–Willi syndrome, attention, quantitative electroencephalogram, alpha power, Conners’ continuous performance test

## Abstract

**Introduction:** Attention problems are frequently observed in patients with Prader–Willi syndrome (PWS); however, only few studies have investigated the severity and mechanisms of attention problems in them. In this study, we aim to evaluate dynamic changes in the quantitative electroencephalographic (EEG) spectrum during attention tasks in patients with PWS.

**Method:** From January to June 2019, 10 patients with PWS and 10 age-matched neurotypical control participants were recruited at Taipei Tzu Chi Hospital. Each participant completed Conners’ continuous performance test, third edition (CPT-3), tasks with simultaneous EEG monitoring. The dynamic changes in the quantitative EEG spectrum between the resting state and during CPT-3 tasks were compared.

**Results:** Behaviorally, patients with PWS experienced significant attention problems, indicated by the high scores for several CPT-3 variables. The theta/beta ratio of the resting-state EEG spectrum revealed no significant differences between the control participants and patients with PWS. During CPT-3 tasks, a significant decrease in the alpha power was noted in controls compared with that in patients with PWS. The attention-to-resting alpha power ratio was positively correlated with many CPT-3 variables. After adjusting for genotype, age, intelligence, and body mass index, the attention-to-resting alpha power ratio was still significantly correlated with participants’ commission errors.

**Conclusion:** This study provides evidence that attention problems are frequently observed in patients with PWS, while attention impairment can be demonstrated by dynamic changes in the quantitative EEG spectrum.

## Introduction

Prader–Willi syndrome (PWS) is a genetically determined multisystem disorder with an estimated prevalence of 1/10,000–1/30,000 individuals (Cassidy et al., 2012). PWS is a well-known genetic obesity syndrome. Hyperphagia and lack of satiety are cardinal symptoms of PWS, which often lead to morbid obesity if the food environment is unsupervised (Miller et al., 2011). Moreover, the characteristic neurobehavioral symptoms of PWS impose severe caregiver burden and stress for families (Kayadjanian et al., 2018; Wong et al., 2021a), with attention problems being highly prevalent in these patients (Gross-Tsur et al., 2001; Wigren and Hansen, 2005; Wong et al., 2021a). Genetic defects of PWS result in widespread developmental abnormalities of brain structures (Manning et al., 2018) and neurotransmitter systems (Luck et al., 2016; Wu et al., 2020), which are strongly associated with the pathogenesis of attention problems (Faraone et al., 2015). In detail, PWS is caused by a loss of expression of imprinted paternally inherited genes on chromosome 15q11.2-q13, including five functional genes (*MKRN3*, *MAGEL2*, *NDN*, *NPAP1*, and *SNURF-SNRPN*) and a cluster of small nucleolar RNA genes (Wong et al., 2020). Among these genes, *MAGEL2* and *NDN* regulate neurite axonal outgrowth through the inhibition of FEZ1/2 degradation and may be related to the pathogenesis of PWS-related neuropsychiatric symptoms (Ehrhart et al., 2018). This speculation is supported by experiments on genetically engineered mouse models of PWS; Magel2-deficient mice have shown defective neuropeptide secretion ability (Chen et al., 2020) and dopaminergic dysfunction (Luck et al., 2016), and Ndn-deficient mice have shown abnormal noradrenergic excitability (Wu et al., 2020). These two catecholamines play important roles in the pathogenesis of attention-deficit/hyperactivity disorder (ADHD) and are also the mechanistic targets of methylphenidate, which is the most widely used medication to treat ADHD (Faraone, 2018).

However, only few studies have investigated the severity and mechanisms of attention problems in patients with PWS (Gross-Tsur et al., 2001; Wigren and Hansen, 2005). Because the pathogenesis of ADHD involves the malfunction of various brain circuits and neuromodulatory systems, scalp electroencephalography (EEG), which represents cortical neuron activity, may reflect disease activity or serve as a neurophysiological biomarker (Newson and Thiagarajan, 2019), such as the increased theta/beta ratio (TBR) of the EEG spectrum in patients with ADHD (Clarke et al., 2020). Although the clinical application of TBR is highly controversial, it was approved by the United States Food and Drug Administration as confirmatory support for the diagnosis of ADHD in patients aged 6–17 years (Gloss et al., 2016; Saad et al., 2018). Except for TBR, the alpha power of the EEG spectrum is also regarded as a biomarker of ADHD and can reflect the level of cortical activity related to the attention status (Sander et al., 2010; Deiber et al., 2020).

In this study, we sought to answer the following questions: 1. How is the severity of attention problems in patients with PWS analyzed? 2. Can EEG be a neurophysiological biomarker of attention problems in patients with PWS? Conners’ continuous performance test third edition (CPT-3) is a widely used performance validity test to assess an individual’s vigilance and sustained attention, which are important factors in the diagnosis of ADHD (Ord et al., 2020). Previous studies have validated that attention-induced cortical activation by CPT-3 tasks can be monitored with brain functional studies, such as quantitative EEG and near-infrared spectroscopy (Nazari et al., 2011; Miao et al., 2017). We proposed the potential of quantitative EEG as a promising neurophysiological tool to assess attention problems. Therefore, our study aimed to evaluate dynamic changes in the quantitative EEG spectrum during attention tasks in patients with PWS.

## Materials and Methods

### Participants

From January to June 2019, the study recruited patients with PWS and age-matched control participants from Taipei Tzu Chi Hospital. All patients with PWS were diagnosed after more than one of the following tests was performed: PWS-specific methylation-specific polymerase chain reaction, fluorescence *in situ* hybridization, or methylation multiplex ligation-dependent probe amplification (Lin et al., 2007). The control participants had no symptoms of ADHD according to the criteria of the Diagnostic and Statistical Manual of Mental Disorders, Fifth Edition (Diagnostic and statistica, 2013). The intelligence of patients with PWS was tested with the Wechsler Intelligence Scale for Children or the Wechsler Adult Intelligence Scale, according to their age at examination. The study was conducted in accordance with the Declaration of Helsinki and was approved by the Local Ethics Committee of Taipei Tzu Chi General Hospital (07-XD-095). Written informed consent was obtained from the parents of all participants prior to enrollment in the study. We performed a power calculation based on the previous study of quantitative EEG in patients with ADHD (White et al., 2005). With a sample size of 10 in each group, we were powered at 90% to detect differences in the magnitude. Therefore, we recruited 10 patients with PWS and 10 control participants in this study.

### Procedure

During the assessment, the participants were seated on an armchair in a silent room. EEG was recorded, and continuous recordings were obtained during the conduction of the entire procedure. The EEG recording protocol was as follows: first session, eyes closed for 3 min; second session, eyes opened for 3 min; third session, performing CPT-3 tasks for 14 min; fourth session, eyes closed with 75-dB noise stress for 3 min; fifth session, eyes opened with 75-dB noise stress for 3 min; and sixth session, performing CPT-3 tasks with 75-dB noise stress for 14 min. In this study, only the EEG recordings of the first and third sessions were analyzed.

**FIGURE 1:**
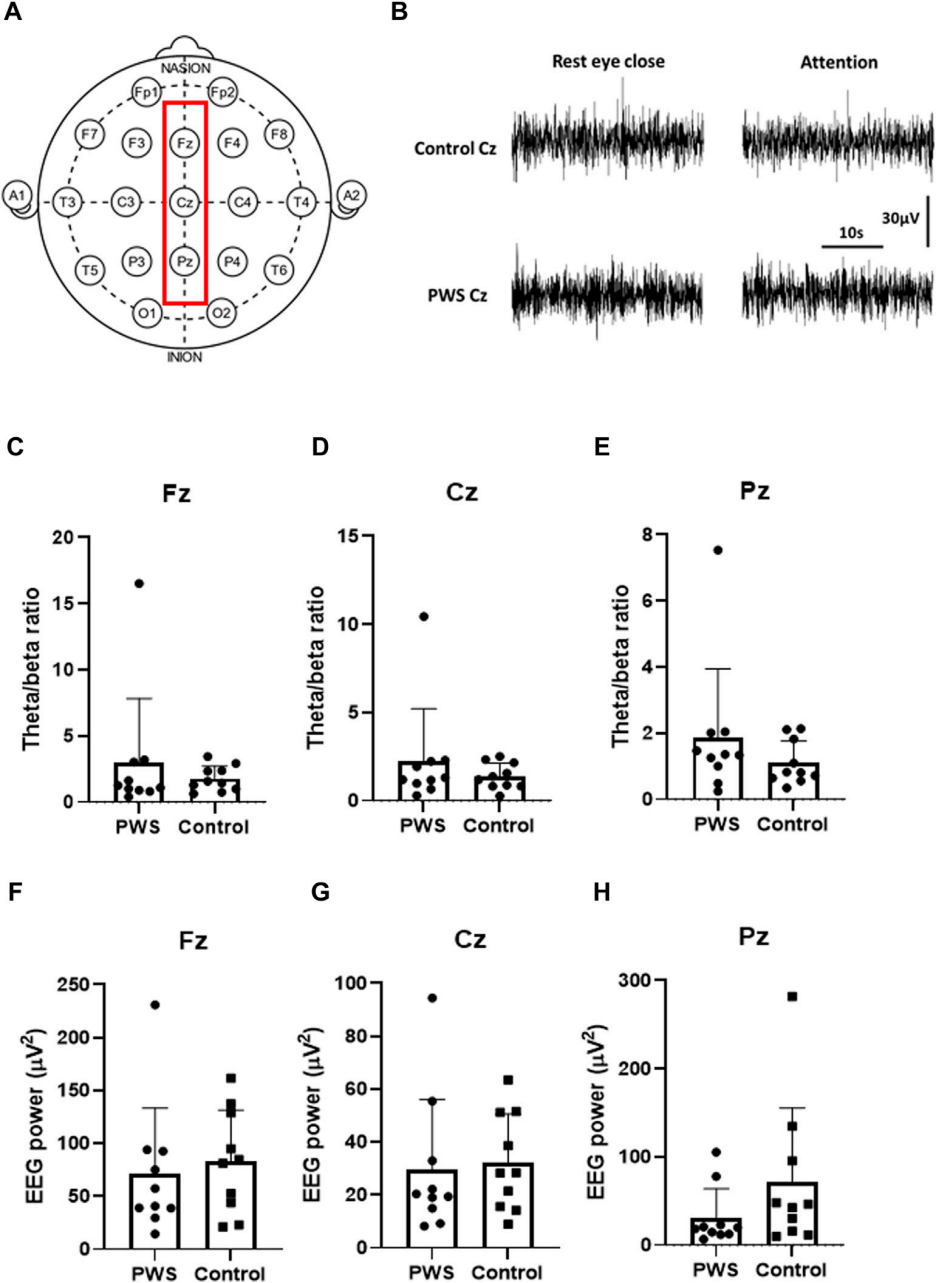
Quantitative electroencephalographic (EEG) analysis during the resting state. **(A)** Schematic representation of the surface EEG of the International 10–20 system. The red square reflects the midline electrodes (Fz, Cz, and Pz) that were analyzed in this study. **(B)** Representative raw traces of Cz EEG in a control participant and a patient with PWS. **(C–E)** Theta/beta ratio of Fz **(C)**, Cz **(D)**, and Pz **(E)** during the resting state revealed no significant differences in patients with PWS and control participants. **(F–H)** 0-50-Hz EEG power of Fz **(F)**, Cz **(G)**, and Pz **(H)** during the resting states showed no significant differences in patients with PWS and control participants.

**FIGURE 2:**
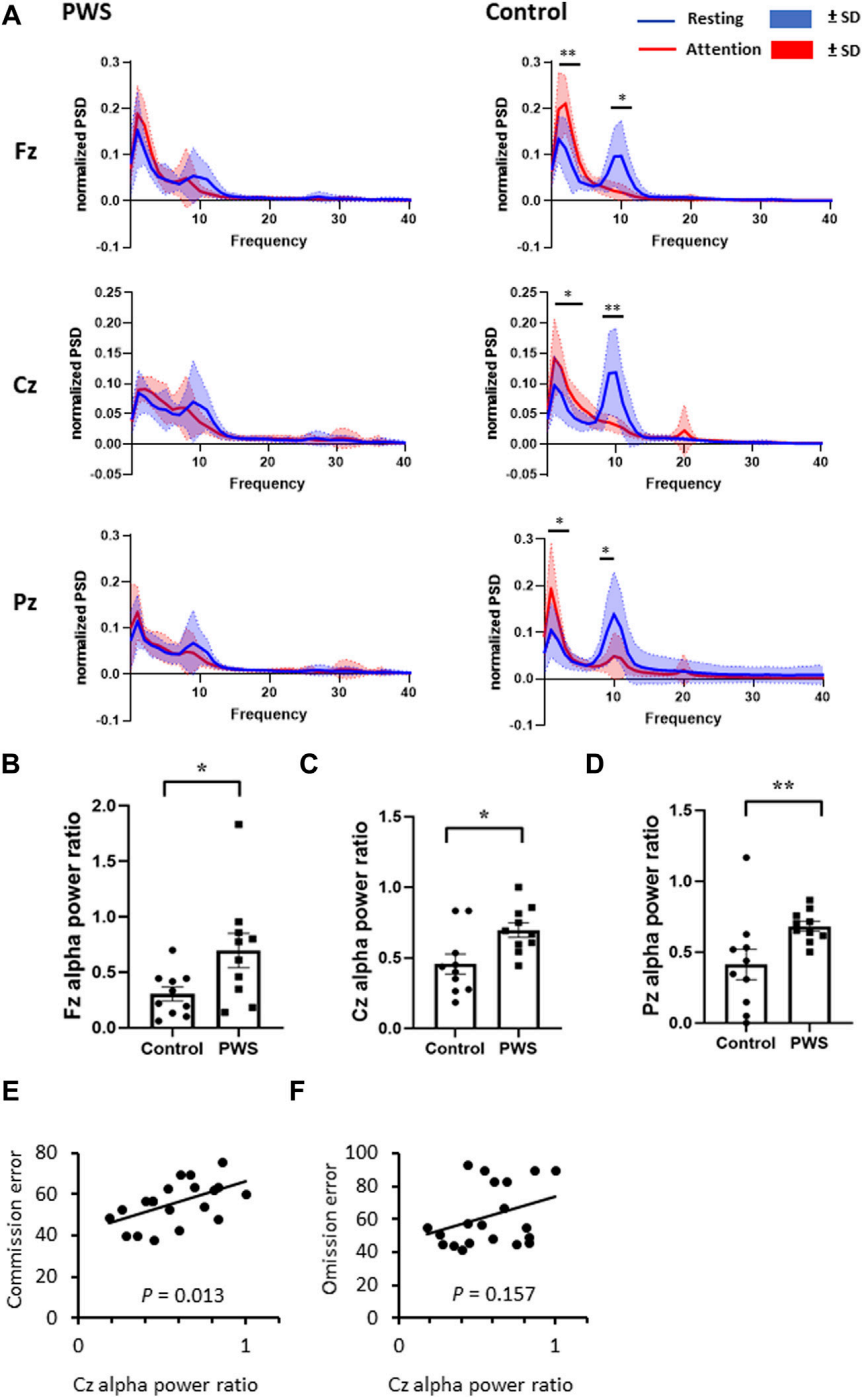
Dynamic changes in the quantitative electroencephalographic (EEG) spectrum between an attention task and the resting state. **(A)** Normalized EEG power spectrum density during attention tasks and resting state in patients with PWS and control participants. The control participants presented with an increased delta-to-theta power and a decreased alpha power while engaging in attention tasks. **(B–D)** The attention-to-resting alpha power ration were smaller in control participants at Fz **(B)**, Cz **(C)**, and Pz **(D)** regions. **(E, F)** For all participants, including patients with PWS and participants, the attention-to resting Cz alpha power ratio positively correlated with the T-scores of commission error **(E)** but not with omission error **(F)**.

We assessed EEG signals from three midline electrodes (Fz, Cz, and Pz; Figure 1A). First, we analyzed the TBR and total EEG power of the EEG recording during rest with the participant’s eyes closed (first session). Next, we analyzed the total EEG power at the midline electrodes. We then normalized the 0–50 Hz EEG power of each participant and assessed the dynamic changes in the normalized EEG power spectrum density (PSD) between the attention task (third session) and resting state (first session) (Figure 2A).

### Continuous Performance Test 3rd Edition

During the 14-min 360-trial administration, the participants were required to respond when any letter appeared, except the non-target letter “X.” During administration, the respondents were asked to press the space bar or mouse button when any target letter is presented on the screen and to inhibit responding when a non-target letter (X) is presented. CPT-3 reflects attention-related problems in individuals aged 8 years and above. CPT-3 yields scores for the following nine variables: detectability, omission errors, commission errors, perseverations, hit reaction time (HRT), HRT standard deviation (SD), variability, HRT block change, and HRT inter-stimulus interval (ISI) change. Detectability is a measure of how well the respondent discriminates non-targets from targets. Omission errors represent the number of targets missed. Commission errors indicate incorrect responses to non-targets. Perseverations indicate responses that are made in less than 100 ms following the presentation of a stimulus. HRT reflects the average response speed. HRT SD indicates the consistency of the response speed to targets for the entire administration duration. Variability also is a measure of consistency of the response speed; however, unlike HRT SD, it is a “within respondent” measure, as it assesses the amount of variability that the respondent shows in 18 separate sub-blocks in relation to their overall HRT SD score. The HRT block change indicates the change in the HRT across blocks. The HRT ISI change reflects changes in the HRT across three inter-stimulus intervals (1, 2, and 4 s). A higher HRT ISI change score indicates that the respondents’ information processing efficiency has declined with longer pauses between stimuli, suggesting a loss of vigilance (Keith Conners et al., 2018).

### Electroencephalography Data Processing

EEG recordings were acquired with Neurofax EEG-1200 (Nihon Kohden, Tokyo, Japan) using Ag/AgCl electrodes at a sampling rate of 200 Hz. A total of 19 electrodes were placed by a trained research technologist, according to the International 10–20 system (Figure 1A). All electrodes were referenced to the ground electrode in the Fpz position during the recording. After visual inspection of artifact rejection, a 60-s EEG recording was retrieved in each session for further analysis. Digitized EEG data were processed offline using in-house MATLAB scripts developed for frequency analyses, which were transformed into frequency domains *via* a PSD function (by using the Welch’s method with the Hanning window, the sampling rate at 200 Hz in a data block of 1 s, administered at a frequency resolution of 1 Hz, and with half of the data overlap in each step), similar to our previous study (Pan et al., 2020; Wong et al., 2021b). We summed the PSD data of the 60-s epoch of each recording session and analyzed in theta (4–7 Hz), alpha (8–12 Hz), and beta (13–25 Hz) frequency bands. We calculated the normalized PSD at a specific frequency by measuring the ratio of the PSD of that specific frequency to the sum of the total PSD of 0–50 Hz.

### Normalized Body Mass Index Z-Score

To adjust for age and sex, the patients’ BMI values were converted into z-scores using age- and sex-specific BMI norms for Taiwan (Chen and Chang, 2010). Each z-score was calculated by subtracting a patient’s BMI from the mean BMI of the patient’s age group and dividing the difference by the SD of the BMI for that age group. Overweight was defined as a BMI z-score of >1 and obesity as a BMI z-score of >2.

### Statistical Analysis

Statistical analyses were performed using SPSS version 19.0 (IBM Corporation, Armonk, NY, United States) for Windows (Microsoft Corporation, Redmond, WA, United States). Descriptive data are presented as mean ± SD. The chi-square test and Mann–Whitney U test were used to compare factors such as age, sex, BMI, BMI z-score, overweight/obesity, T-score of CPT-3 variables, TBR, EEG power, and alpha power ratio between patients with PWS and control participants. The independent *t*-test was used to compare the normalized EEG power of each frequency between patients with PWS and control subjects. Spearman’s correlation analysis was used to assess the relationship between the alpha power ratio and the T-scores of CPT-3 variables. Multivariate stepwise linear regression models were used to explore the relationship between variables, including the commission error T-score, the omission error T-score, the genotype, age, the BMI z-score, intelligence, and the attention-to-resting Cz alpha power ratio. Two-sided *p* values of <0.05 were considered to be statistically significant.

## Results

### Demographic and Behavioral Data of Participants

The demographic data of the participants are summarized in Table 1. The age of the patients with PWS and control participants was 18.5 ± 8.0 and 19.8 ± 5.8 years, respectively (*p* = 0.519, Mann–Whitney U test). Overall, 7 patients with PWS had moderate-to-severe intellectual disabilities, and although 7 of the 10 patients with PWS were overweight or obese, the BMI and BMI z-scores of patients with PWS and control participants showed no significant differences (Table 1). Although the mean IQ of patients with PWS was 53, they can follow CPT-3 instructions and complete the test. The T-scores of CPT-3 variables in patients with PWS and control participants are shown in Table 2. Patients with PWS had significantly higher T-scores for detectability, omission, commission, preservation, HRT, HRT SD, and variability, indicating a significant attention impairment in patients with PWS.

**TABLE 1:** Demographic data of patients with Prader–Willi syndrome and control subjects.

	PWS (n = 10)	Control (n = 10)	*p*-value
	Baseline characters		
Age (years)	18.5 ± 8.0	19.8 ± 5.8	0.519^a^
Sex (M/F)	4/6	5/5	0.653^b^
Genotype (Del/non-Del)	6/4	NA	—
Moderate-to-severe ID	7	0	<0.001^b^ ^**^
BMI	25.7 ± 5.9	23.6 ± 3.5	0.406^a^
BMI z-score	2.0 ± 2.1	0.8 ± 0.9	0.307^a^
Overweight/obesity	7	4	0.178^b^

Values are expressed as mean ± SD.

Abbreviations: PWS, Prader–Willi syndrome; ID, intellectual disability; Del, deletion type; non-Del, non-deletion type; and BMI, body mass index.

a Mann-Whitney test.

b Chi-square test.

** *p* < 0.01.

**TABLE 2:** Conner’s CPT scores of patients with PWS and control subjects.

CPT T score	PWS (n = 10)	Control (n = 10)	*p*-value
Detectability	67.6 ± 11.2	51.4 ± 8.6	0.004^a^ ^**^
Omission	74.4 ± 18.9	49.3 ± 5.7	0.007^a^ ^**^
Commission	60.9 ± 18.3	50.9 ± 9.5	0.043^a^ ^*^
Perseveration	78.7 ± 7.3	48.8 ± 3.2	<0.001^a^ ^**^
HRT	69.1 ± 17.1	48.9 ± 7.4	0.003^a^ ^**^
HRT standard deviation	75.7 ± 18.4	46.3 ± 5.8	0.002^a^ ^**^
Variability	71.3 ± 16.8	46.7 ± 5.3	0.003^a^ ^**^
HRT block change	37.5 ± 19.3	52.3 ± 9.9	0.075^a^
HRT ISI change	61.1 ± 15.8	53.0 ± 6.0	0.315^a^

Values are expressed as mean ± SD.

Abbreviations: CPT, continuous performance test; PWS, Prader–Willi syndrome; HRT, hit reaction time; and ISI, inter-stimulus intervals.

a Mann–Whitney test.

* *p* < 0.05, **p < 0.01.

### Dynamic Changes in the Quantitative Electroencephalography Spectrum

TBR and total EEG power of the EEG spectrum were analyzed during rest with eyes closed. Although patients with PWS had significant attention impairment documented by CPT-3, the TBR at the midline electrodes showed no significant differences in patients with PWS and control participants (Figure 1C–E). Furthermore, the total EEG power at the midline electrodes also revealed no differences between patients with PWS and control participants (Figure 1F–H), but the variation within the study groups was large. Therefore, we normalized the 0–50 Hz EEG power of each participant to compare the dynamic changes of quantitative EEG between the two study groups.

During CPT-3, control participants had significantly increased delta and theta EEG power values and decreased alpha EEG power value over the midline electrodes; in contrast, the attention task did not change the PSD in patients with PWS (Figure 2A). Given that the dynamic change in PSD in the alpha frequency range is significant, we divided the alpha power PSD during the attention task (third session) by the alpha PSD during the resting state (first session) of each participant. The attention-to-resting alpha power ratio was significantly lower in control participants than in patients with PWS at Fz, Cz, and Pz (Figure 2B–D). Therefore, we designated the attention-to-resting Cz alpha power ratio as a neurophysiological marker of attention and examined its correlation with T-scores of the CPT-3 variables. For all participants, the Cz alpha power ratio positively correlated with multiple variables, including detectability (Spearman’s coefficient 0.524, *p* = 0.018), commission errors (Spearman’s coefficient 0.547, *p* = 0.013, Figure 2E), perseveration (Spearman’s coefficient 0.534, *p* = 0.015), HRT SD (Spearman’s coefficient 0.524, *p* = 0.018), variability (Spearman’s coefficient 0.506, *p* = 0.027), and HRT block change (Spearman’s coefficient 0.472, *p* = 0.036), which further confirmed that cortical activation during the attention task can be monitored by the decrease in alpha power PSD. However, the omission error T-score was not correlated with the attention-to-resting alpha power ratio (Spearman’s coefficient 0.329, *p* = 0.157, Figure 2F), indicating that commission and omission errors possibly presented with different brain mechanisms.

Given that the participants’ genotype, age, intelligence, and BMI possibly affected their performance in the behavior task, we explored the relationship between the T-score of the commission and omission errors with the aforementioned variables using multivariate stepwise linear regression models. After adjusting for age, genotype, intelligence, and BMI z-score, the Cz alpha power ratio was the only factor that significantly affected the commission error T-score (*B* = 25.04, *p* = 0.016, Table 3). Conversely, for omission errors, the participants’ intelligence was the only factor with significant correlation (*B* = 14.41, *p* < 0.001, Table 4).

**TABLE 3:** Multivariate stepwise linear regression analysis coefficients of the association between the commission error T-score and attention-to-resting Cz alpha power ratio.

Variables	Coefficient (*B*)	*t*	*p*-value
Cz alpha power ratio	25.04	2.67	0.016^*^
Genotype^a^	0.27	1.15	0.265
Age	−0.18	−0.91	0.374
Intelligence^b^	0.27	1.15	0.267
BMI z-score	0.06	0.28	0.786

a Control = 0, PWS = 1.

b Normal intelligence = 0, mild intellectual disability = 1, and moderate-to-severe intellectual disability = 2.

Abbreviations: BMI, body mass index; PWS, Prader–Willi syndrome.

* *p* < 0.05.

**TABLE 4:** Multivariate stepwise linear regression analysis coefficients of the association between the omission error T-score and attention-to-resting Cz alpha power ratio.

Variables	Coefficient (*B*)	*t*	*p*-value
Cz alpha power ratio	−0.07	−0.34	0.739
Genotype^a^	0.13	0.27	0.788
Age	0.06	0.37	0.715
Intelligence^b^	14.41	4.38	<0.001^**^
BMI z-score	0.09	0.54	0.788

a Control = 0, PWS = 1.

b Normal intelligence = 0, mild intellectual disability = 1, and moderate-to-severe intellectual disability = 2.

Abbreviations: BMI, body mass index; PWS, Prader–Willi syndrome.

* *p* < 0.05.

## Discussion

In this study, we assessed attention problems in patients with PWS using behavioral and EEG approaches. Behaviorally, patients with PWS had significantly higher T-scores for CPT-3 variables. Although we failed to find a difference in the TBR of patients with PWS and control participants, the dynamic changes in the EEG spectrum for attention tasks were significantly impaired in patients with PWS. The attention-to-resting alpha power ratio significantly correlated multiple attention performance variables of CPT-3, and after adjusting for the participants’ genotype, age, intelligence, and BMI, the alpha power ratio still significantly correlated with commission errors. Taken together, our study provides evidence that impaired attention can be frequently observed in patients with PWS; moreover, the impairment in attentive tasks can be demonstrated by dynamic changes in the EEG spectrum. The attention-to-resting alpha power ratio could potentially serve as a neurophysiological marker for attention.

In this study, we used the behavior task (CPT-3) to demonstrate attention problems in patients with PWS. Although ADHD is a common comorbidity in patients with PWS, the diagnosis discussed in previous studies has mostly relied on parent-reported questionnaires (Wigren and Hansen, 2005; Wong et al., 2021a). Our study escalates the behavioral evidence of ADHD in patients with PWS and opens the window to study genotype–phenotype correlations between ADHD and specific genetic dysfunction of PWS, which may improve treatment. Although these two groups of participants behaved significantly different in attention tasks, which is consistent with the findings of previous studies reporting that TBR was inadequately accurate for a diagnosis of ADHD (Kiiski et al., 2020), the TBR of the resting-state EEG failed to distinguish patients with PWS and control subjects in our study. In contrast, while engaging in attention tasks, control participants showed a 60–70% decrease in the alpha power of the EEG spectrum, and the patients with PWS showed a decrease of 30% only. A decrease in the alpha power in neurotypical children during the CPT task has been reported in a study conducted by Nazari et al.; however, the CPT task in that study induced an increase in the alpha power of patients with ADHD (Nazari et al., 2011), which is in contrast with our findings. This contradictory experimental result may be explained by our different experimental designs. In Nazari et al.’s study, they compared the alpha power during the attention task with the EEG recorded during resting with eyes opened, which significantly suppressed the alpha power in the parieto-occipital regions. In our study, we chose the eye-closed condition as the baseline, which showed a larger alpha power. Our study produced results demonstrating that engaging in attention tasks could reduce the alpha power of the EEG spectrum in non-ADHD participants. Moreover, we discovered a positive correlation between an individual’s decreased alpha power and attention task performance. The alpha power ratio of attention-to-resting could potentially be a new physiological marker for attention problems and could be applied to clinical practice or neurofeedback training programs.

Most patients of PWS in this study had moderate-to-severe intellectual disability, which is a possible confounder of quantitative EEG abnormalities and impaired CPT-3 scores. In our study, patients with PWS and control participants had a similar resting-state quantitative EEG spectrum, implying that the default neural oscillations of these two groups did not alter by their baseline intellectual differences. This finding is supported by a previous study reporting that inattentive children with low or normal intelligence shared similar quantitative EEG profiles during resting state (Clarke et al., 2006). While engaging in attentive tasks, patients with PWS had defective alpha-band oscillation suppression which is a neurological marker of selective attention (Foxe and Snyder, 2011). Therefore, we considered that the defective alpha suppression of patients with PWS was related with attention deficit rather than cognitive impairment. Intelligence indeed would influence the results of attention tasks. The previous study demonstrated that children with superior intelligence would have better scores in CPT (Park et al., 2011); therefore, the impaired CPT performance of patients with PWS cannot absolutely refer to ADHD and was partly because of their low intelligence. Because attention is an important predictor of intelligence (Schweizer and Moosbrugger, 2004), it is difficult to find a control group of patients with only an intellectual disability and no symptoms of ADHD. In addition, both intellectual disability and attention problems were stressors for families of PWS (Wong et al., 2021a); therefore, abnormal CPT-3 performance of patients with PWS indicated their significant functional deficits and highlighted the importance of dedicated medical and social support.

This study had some limitations. First, owing to the small sample size of this study, the differences in TBR between patients with PWS and control subjects cannot possibly be demonstrated. Second, given the diverse age of the participants between teenager and young adult, we cannot assess the severity of ADHD using a standardized questionnaire, such as the Child Behavior Checklist or the Swanson, Nolan, and Pelham Rating Scale IV. Therefore, there is a range of difficulty if the patients with PWS in this study can be classified as ADHD. However, with the modest sample size of this study, we revealed that patients with PWS showed significant impairment in attention tasks. Another unexplored issue is the influence of intellectual disability on dynamic changes in the EEG spectrum. Although the multivariate linear regression model revealed that the attention-to-resting alpha power ratio and intellectual disability had different roles in the participants’ commission and omission errors, the underlying mechanisms and interactions of attention problems and intellectual disability and their influence on dynamic changes in the EEG spectrum remain to be assessed. Large-scale studies are warranted to facilitate the clinical utilization of the attention-to-resting alpha power ratio as a physiological marker of attention.

## Conclusion

In conclusion, patients with PWS experienced significant problems with attention tasks, and this deficit was demonstrated by the dynamic changes in the EEG spectrum. The decreased alpha power during attention tasks could potentially serve as a physiological biomarker of attention, which could be applied to clinical practice or to develop neurofeedback training. The assessment and treatment experience in patients with PWS could be further applied to other patients with idiopathic ADHD.

## Data Availability

The original contributions presented in the study are included in the article/Supplementary Material, further inquiries can be directed to the corresponding author.
